# Fibrinogen levels in relation to colorectal cancer onset: A nested case-cohort study from the Moli-sani cohort

**DOI:** 10.3389/fcvm.2022.1009926

**Published:** 2022-10-13

**Authors:** Roberta Parisi, Teresa Panzera, Laura Russo, Sara Gamba, Amalia De Curtis, Augusto Di Castelnuovo, Marina Marchetti, Chiara Cerletti, Anna Falanga, Giovanni de Gaetano, Maria Benedetta Donati, Licia Iacoviello, Simona Costanzo, the Moli-sani Study Investigators

**Affiliations:** ^1^Department of Medicine and Health Sciences, University of Molise, Campobasso, Italy; ^2^Department of Epidemiology and Prevention, IRCCS Neuromed, Pozzilli, Italy; ^3^Division of Immunohematology and Transfusion Medicine, Hospital Papa Giovanni XXIII of Bergamo, Bergamo, Italy; ^4^Mediterranea Cardiocentro, Naples, Italy; ^5^Department of Medicine and Surgery, University of Milan Bicocca, Monza, Italy; ^6^Department of Medicine and Surgery, Research Center in Epidemiology and Preventive Medicine (EPIMED), University of Insubria, Varese, Italy

**Keywords:** fibrinogen, colorectal cancer, inflammation, hemostasis, atherosclerosis

## Abstract

**Background:**

Patients with cancer are commonly characterized by abnormalities in laboratory coagulation tests, underlying a subclinical hypercoagulable condition. Due to the involvement of the hemostatic system in cancer patients, some of its biomarkers, such as fibrinogen, could be a useful tool in predicting cancer risk. We performed a case-cohort study to evaluate the relationship among fibrinogen levels and colorectal cancer (CRC).

**Methods:**

In the framework of Moli-sani Study (*N* = 24,325, enrolled 2005–2010) a subcohort of 1,290 individuals (55.0% women; mean age 55.0 ± 12.0 years) was selected and compared with 126 CRC cases identified during a follow-up of 4.3 years. Incident cases of colorectal cancer were ascertained by direct linkage with hospital discharge forms according to the International Classification of Disease (ICD-9-CM) codes: 153–154. Events were validated through medical records and confirmed by histological reports. Fibrinogen levels were measured in frozen citrated plasma samples. Hazard Ratio (HR) and 95% confidence interval (CI), adjusted by relevant covariates were estimated by a Cox regression model using Prentice method.

**Results:**

Individuals with levels of fibrinogen ≥400 mg/dL had a higher hazard to develop colorectal cancer when compared to those with lower levels after adjustment for sex and age (HR: 1.81; 95% CI 1.12–2.92). Additional adjustment for CRC family history, income, physical activity, diabetes medication and hypercholesterolemia did not modify the result (HR: 1.91; 95% CI 1.15–3.17). Analyses stratified by age and sex showed a most evident association in elderly (HR: 2.30; 95% CI: 1.10–4.81) and in women (HR: 2.28; 95% CI: 1.08–4.81). Sensitivity analyses confirmed the main findings, showing independence from a potential role of confounding by a large panel of biomarkers, including inflammation and hemostasis factors.

**Conclusion:**

Our results, based on a case-cohort study from a general adult population apparently free from any cancer during the recruitment, showed that fibrinogen levels ≥400 mg/dL were positively and independently associated with CRC, suggesting that this glycoprotein could be a potential biomarker for this type of cancer and supporting the “common soil hypothesis” in the pathophysiology of cardiovascular disease and tumors.

## Introduction

Colorectal cancer (CRC) is one of the most prevalent tumors worldwide, representing 10% of both the most commonly diagnosed and of the leading cause of cancer death (1, 2).

Several pieces of information are available on the risk to develop this oncological disorder. The probability of suffering from CRC can be associated with a wide range of factors, such as some clinical conditions (inflammatory bowel disease, ulcerative colitis and Crohn's syndrome) (3–5), family history (6, 7) and lifestyle habits (unhealthy diet, abuse of alcoholic beverage, physical inactivity and smoking habits) (1, 8).

Despite the improvements achieved in health systems and the implementation of screening programs have positively affected the impact of this disease in the population worldwide, the number of CRC-related deaths and of new cases still remains high (2). In addition, it has been estimated by WHO that the mortality rate of this type of malignancy is predicted to increase sharply in the next 40 years (9).

Considerable evidence has supported the so called “common soil” hypothesis in the pathogenesis of both cardiovascular diseases (CVD) and different types of cancer (especially hormone-dependent and gastrointestinal); this means that these two conditions share some pathophysiological mechanisms and risk factor profiles (10–15). In this context, several components of the hemostatic system have been proposed as potential biomarkers for both (16–20).

In particular, fibrinogen might be a promising tool because of its role in both hemostatic and inflammatory processes. Fibrinogen is a glycoprotein synthesized by the liver and in healthy individuals, its plasma levels range from 200 to 400 mg/dL, with a normal half-life of 3–5 days (17, 20). As activating factor of the clotting process, fibrinogen plays a key role in atherosclerosis (21) and in the pathophysiology of coronary artery disease and CVD (22).

Additionally, fibrinogen is also involved in the inflammatory response (23). It is an acute phase reactant and, during episodes of inflammation, its synthesis is enhanced, making it a biomarker of systemic inflammation (23).

The role of this coagulation protein has been widely investigated, in cancer clinical settings. Indeed, elevated plasma levels of pre-operative fibrinogen have been associated with a poor prognosis of overall/disease free survival, a worst response to therapy and tumor size and extension of tumor invasion in gastrointestinal and, in particular, colorectal cancer (24–30). On the other hand, nowadays, the only study investigating the possible relationship of fibrinogen with cancer in the setting of a general population, is represented, by the work of Allin et al. showing that a simultaneous elevation of levels of inflammatory markers (fibrinogen, C-reactive protein and leukocyte count) was associated with an increased risk of lung, breast and colorectal cancer, with a stronger effect in the first few years of follow-up (31).

In order to improve CRC prevention and decrease its incidence, it is fundamental to identify novel predictive biomarkers for CRC (8, 32).

For this reason, we performed a nested case-cohort study to evaluate the association between plasma levels of fibrinogen and CRC in the framework of the Moli-sani study population.

## Materials and methods

### Study population and study design

The Moli-sani study is an ongoing prospective cohort study that enrolled 24,325 individuals (51.9% women, aged ≥35 years) randomly recruited from the general population of Molise region in Southern-central Italy between 2005 and 2010. Exclusion criteria were pregnancy at the time of recruitment, disturbances in understanding or willingness, current poly-traumas or coma, refusal to sign the Informed Consent form (33). The Moli-sani study complies with the Declaration of Helsinki and was approved by the Catholic University Ethical Committee, Rome, Italy. All the individuals enrolled provided written informed consent. During the baseline visit, trained researchers administered questionnaires about personal and family medical history, physical activity, dietary habits, risk factors for cardiovascular and cancer diseases, socioeconomic status and measured blood pressure and anthropometry. Additionally, blood samples were obtained at the baseline visit by venous puncture between 07:00 and 09:00 a.m. from individuals who had fasted overnight and had refrained from smoking for at least 6 h (33). Blood samples were rapidly processed and stored in liquid nitrogen (−196°C) at the Neuromed Biobanking Centre (https://www.neuromed.it/ricerca/biobanking-centre/) during the same day of recruitment visist. Details of the Moli-sani study have been described in Supplementary materials and in previous publications (33, 34).

One hundred and twenty-six cases of CRC were identified among the individuals of the Moli-sani cohort, during a median follow-up of 4.3 years (35). The subcohort was randomly extracted from the Moli-sani cohort (parent cohort) and included 1,290 individuals without a history of any cancer at baseline or incident no-CRC cancer events, or missing values for fibrinogen. However, the subcohort included eight individuals who had developed CRC, due to the random selection from the parent cohort.

### Incident colorectal cancer assessment

Incident cases of CRC were ascertained by direct linkage with hospital discharge forms according to the ICD-9-CM codes: 153 (malignant neoplasm of colon) and 154 (malignant neoplasm of rectum, rectosigmoid junction and anus). The validation of CRC events was performed through medical records and was confirmed by the histological reports. Additionally, fatal cases of CRC were assessed by direct linkage with the regional register of cause of death (ReNCaM registry), when then underlying cause of death presented ICD-9 codes as 153 or 154. For the present case-cohort study, the end of follow-up was 31^st^ December 2011.

Information on CRC stage and grade were collected through the histological reports (missing data 15.9 and 23.8%, respectively). The CRC stage and grade were classified according to the 8th version of the AJCC TNM classification system (36).

### Plasma fibrinogen measurements

Between July 2014 and March 2017, frozen citrated plasma samples of about 2,500 Moli-sani participants selected for the case-cohort study (1000 cases of any cancer, including CRC and 1,500 subcohort individuals), were express-shipped in several batches on dry ice to the Hemostasis and Thrombosis Laboratory of the Division of Immunohematology and Transfusion Medicine, Papa Giovanni XXIII Hospital in Bergamo. Fibrinogen levels were measured, in frozen citrated plasma samples, with a test based on the Clauss method, on the coagulation system ACL TOP 500 (Q.F.A. Thrombin; Werfen). Quality controls included high and low plasma samples, provided by the kit, and a home-made normal pool plasma. Regarding the latter, the CVs for normal and abnormal value were 6.1 and 4.3% for intra- and 6.7 and 5.1% for inter-assay, respectively.

Even if the latter procedure could theoretically influence the laboratory data, it has been documented that freezing and long-term storing seems to have minimal effects on fibrinogen levels and is irrelevant for clinical interpretation (37).

### Statistical analysis

The normality of continuous variables was assessed by the Shapiro–Wilk test and confirmed graphically. Normally distributed data are presented as mean and standard deviation (SD), skewed as median and interquartile range (IQR), and categorical variables as frequencies.

Fibrinogen levels were classified into quartiles (based on the distributions in the subcohort) or according to hyperfibrinogenemia defined as fibrinogen levels ≥400 mg/dL.

To estimate the relationship among fibrinogen and CRC, Cox proportional-hazard regression modified according to the Prentice method was used, with age as the underlying time scale (38). In the counting processes, age was the underlying time variable with “entry time” defined as age at baseline and “exit time” as age at cancer event or censoring.

Because of the positive skewness of the fibrinogen distribution, hazard ratios (HRs) were also calculated for 1 SD increment of natural log transformed levels of fibrinogen (SD = 0.28 mg/dL).

We fitted a minimally adjusted model with age and sex as covariates (model 1) and one multivariable model [model 2: CRC family history, income, physical activity (tertiles), diabetes medication and hypercholesterolemia]. To build the final multivariable model used to estimate hazard ratios, we identified variables associated with both outcome and exposure (fibrinogen in quartiles) at a *P*-value < 0.20 in age- and sex-adjusted model and then included all such covariates in a full model.

We conducted pre-established stratified analyses according to age at baseline (35–65 years and ≥65 years) and sex, to test a possible modification effect of these conditions. P for interaction between fibrinogen (< or ≥400 mg/dL) and the potential effect modifiers (age, sex) was tested adding corresponding cross-product terms in the model. We also performed subgroup analyses according to anatomical subsite [colon (ICD-9-CM 153) and rectum (ICD-9-CM 154)] and age at diagnosis [early (<60 years) and late (≥60 years)].

Sensitivity analyses were performed after excluding a) individuals with CRC family history, b) CRC events that occurred during the first 12 months of follow-up (the latter was decided to reduce the likelihood of CRC status already in progress before fibrinogen measurement) and c) stratifying by CRC stage and grade.

Finally, we tested the robustness of our findings repeating the main analysis using a case-control approach. Eight CRC cases counted in the subcohort were deleted from the control group (N Cases = 126: N Control = 1,282) and a multivariable logistic regression analysis was performed.

Due to key role of fibrinogen in both the coagulation cascade and the inflammatory responses, we explored if the studied association could be influenced by other inflammatory or hemostasis biomarkers. Additionally, the potential confounding effect of a large panel of biomarkers found to be associated with CRC in previous studies (31, 39–42) was also investigated.

SAS/STAT software, version 9.4, was used for the statistical analyses (SAS Institute Inc., Cary, NC, USA) (43).

## Results

In the Moli-sani subcohort (*N* = 1290, 55.0% women, mean age 55.0 ± 12.0 years) the median value of plasma fibrinogen levels was 292 mg/dL (IQR = 248–348) and the 9.6% had fibrinogen ≥400 mg/dL (Table 1).

**Table 1:** Baseline characteristics in the subcohort (*N* = 1,290) and cases (*N* = 126) according to levels of fibrinogen < or ≥400 mg/dL.

	**Subcohort** ***N*** = **1,290**			**Cases** ***N*** = **126**		
	**Fibrinogen <400 mg/dL**	**Fibrinogen ≥400 mg/dL**	***P*-value**	**Fibrinogen <400 mg/dL**	**Fibrinogen ≥400 mg/dL**	***P*-value**
***N*** **(%)**	1,166 (90.4)	124 (9.6)		100 (79.4)	26 (20.6)	
**Fibrinogen**, *mg/dL*	284 (244–328)	444 (421–482)		296 (242–336)	462 (408–488)	
**Men**	44.9	49.2	0.47	64.0	57.7	0.43
**Age**, *years*	54.6 ± 11.9	59.5 ± 11.6	<0.0001	63.9 ± 9.7	68.2 ± 9.7	0.039
**Age** **≥65 Years**	20.9	33.1	0.0025	49.0	61.5	0.24
**CRC family history**	4.0	3.2	0.68	7.0	7.6	0.84
**Residence**			0.25			0.24
Rural	34.1	27.4		25.0	11.5	
Urban	65.9	72.6		75.0	88.5	
**Education**			0.26			0.34
Up to lower secondary school	51.1	50.4		64.0	61.5	
High school or higher	48.9	49.6		36.0	38.5	
**Income**			0.33			0.52
<40,000 *e*/year	57.7	50.8		63.0	73.1	
≥40,000 *e*/year	11.7	14.5		15.0	3.9	
Not responders	30.6	34.7		22.0	23.1	
**Obesity**, *BMI ≥ 30 kg/m^2^*	28.6	54.0	<0.0001	32.0	42.3	0.39
**Abdominal obesity**	41.9	63.7	<0.0001	49.0	61.5	0.48
**Current smoking**	23.3	25.8	0.17	19.0	19.2	0.51
**Physical activity**, ***MET-h/d***			0.047			0.43
Low	33.8	37.1		22.0	30.8	
Medium	33.0	25.8		36.0	42.3	
High	33.2	37.1		42.0	26.9	
**History of CVD**	5.6	12.2	0.070	8.2	0.0	-
**Diabetes medication**	3.7	10.7	0.0082	11.5	20.0	0.34
**Hypercholesterolemia**	29.8	30.0	0.67	27.3	26.9	0.98
**Hypertension**	55.1	72.4	0.092	70.7	92.3	0.068

CRC, colorectal cancer; BMI, body mass index; CVD, cardiovascular disease.

Values are reported as means with standard deviation for continuous variables and percentage for categorical variables. Median values and IQR are reported for fibrinogen. P-values are adjusted for age and sex.

The socio-demographic and clinical characteristics of the population study have been shown in Table 1 and Supplementary Table S1. Higher prevalence of men, adults aged ≥65 years, current smokers, individuals living in urban areas, use of diabetes medication, history of hypertension or CVD was found in the group with fibrinogen levels ≥400 mg/dL (Table 1).

**Figure 1:**
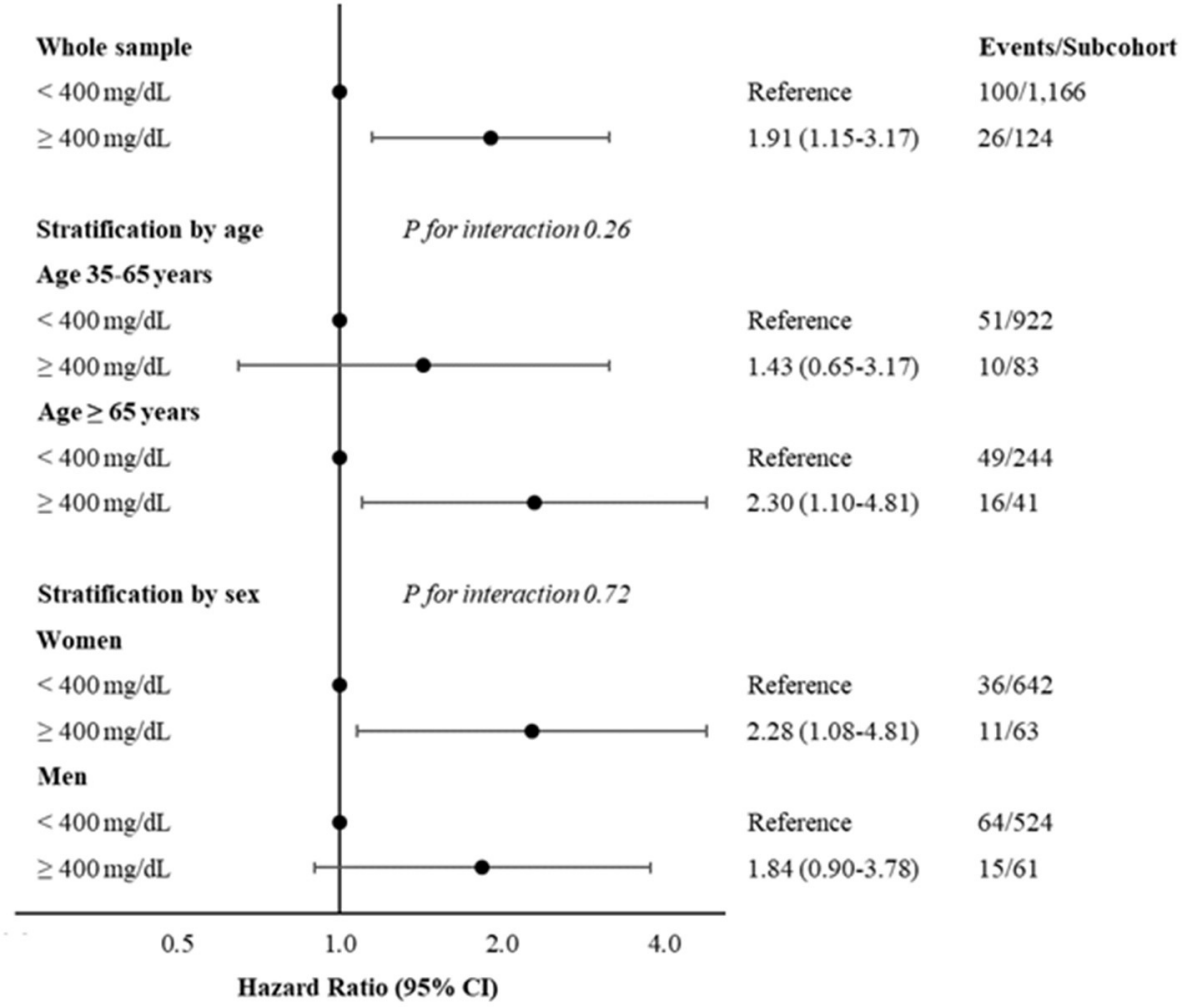
Hazard ratios and 95% CI, for developing colorectal cancer according to levels of fibrinogen < or ≥400 mg/dL, in whole sample and stratified by age (35–65 years and ≥65 years) or sex. Multivariable model adjusted for age (when not stratified by), sex (when not stratified by), CRC family history, income, physical activity, diabetes medication and hypercholesterolemia. CRC, colorectal cancer.

No associations with CRC were observed either according to fibrinogen quartiles, or for 1 SD increase of log-transformed fibrinogen (Supplementary Table S2). On the other hand, higher levels of plasma fibrinogen (≥400 mg/dL) were associated with a higher hazard of CRC, HR: 1.81, 95% CI: 1.12–2.92 in age and sex adjusted model (Table 2, Model 1) and HR: 1.91, 95% CI: 1.15–3.17 in the multivariable model (Table 2 and Figure 1, Model 2).

**Table 2:** HR (95% confidence interval) for developing colorectal cancer according to levels of fibrinogen < or ≥400 mg/dL.

	**Fibrinogen**		
	**<400 mg/dL**	**≥400 mg/dL**	***P*-value**
**Events/Subcohort**	100/1,166	26/124	
**HR crude**	Reference	1.77 (1.09–2.85)	0.020
**HR^a^**	Reference	1.81 (1.12–2.92)	0.015
**HR^b^**	Reference	1.91 (1.15–3.17)	0.012

a Model 1: adjusted for age and sex.

b Model 2: adjusted for age, sex, CRC family history, income, physical activity, diabetes medication and hypercholesterolemia.

Supplementary Table S3 reports associations of higher levels of fibrinogen with CRC, including one by one demographic, lifestyle and clinical variables in the model adjusted for age and sex.

When the case-control study design has been considered, we observed that high levels of fibrinogen led to higher odds of CRC (OR: 1.93; 95% CI 1.16–3.21, Model adjusted for age, CRC family history, income, physical activity diabetes medication and hypercholesterolemia) (Supplementary Table S4).

Figure 1 and Supplementary Table S5 report also stratified analyses by age classes at the baseline (35–65 and ≥65 years) and sex, showing a most evident association in elderly (HR: 2.30; 95% CI: 1.10–4.81) and in women (HR: 2.28; 95% CI: 1.08–4.81). However, there was not a significant interaction for both stratified analyses (P for interaction 0.26 and 0.72 for age and sex strata, respectively).

**Figure 2:**
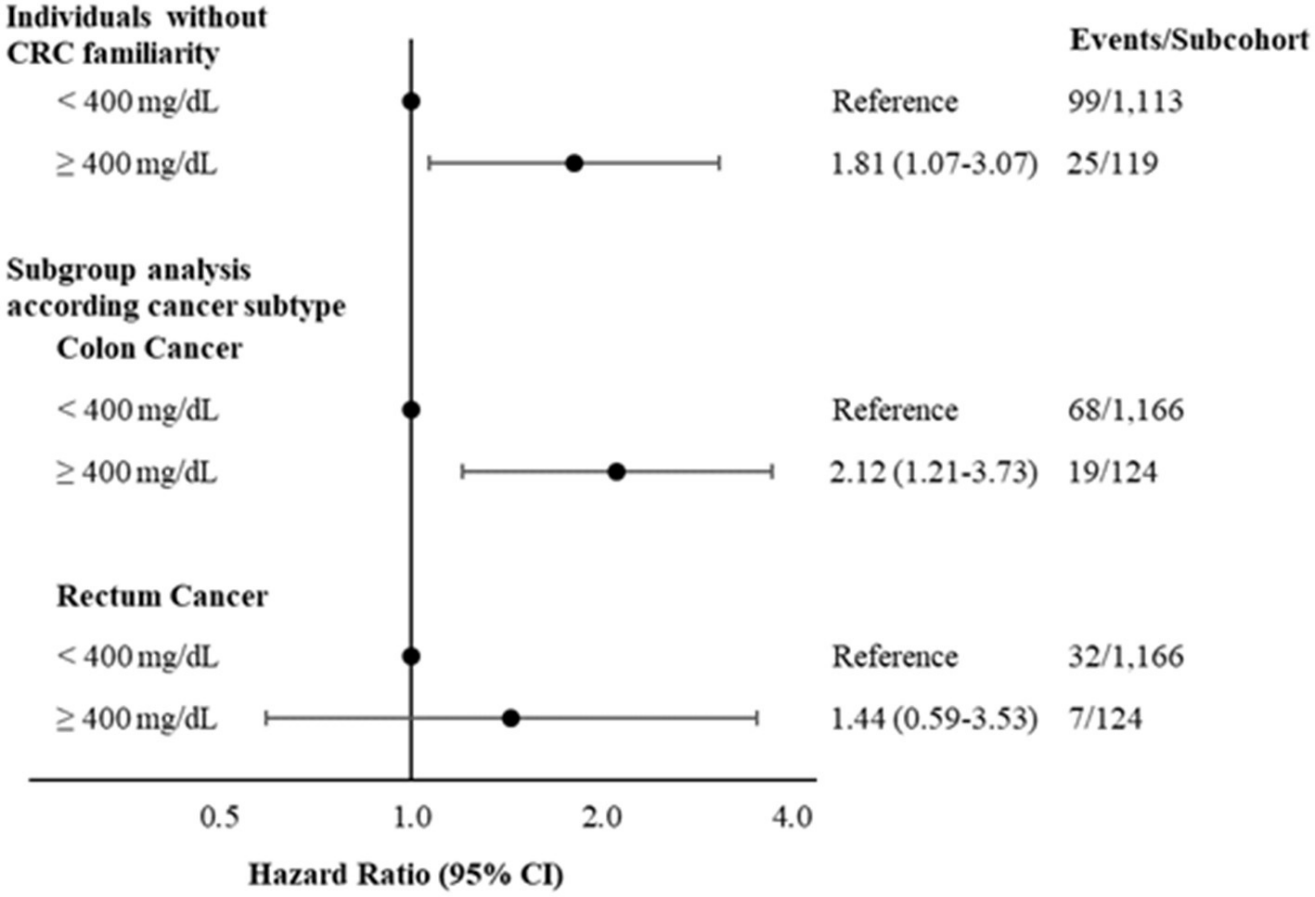
Hazard ratios and 95% CI, for developing colorectal cancer according to levels of fibrinogen < or ≥400 mg/dL excluding individuals with CRC family history and considering different CRC subtypes. Multivariable model adjusted for age, sex, CRC family history (when not stratified by), income, physical activity, diabetes medication and hypercholesterolemia. CRC, colorectal cancer.

Sensitivity analyses excluding individuals with CRC family history confirms the main finding (HR: 1.81; 95% CI: 1.07–3.07) (Figure 2).

Analyses according to CRC subtypes suggest that higher levels of fibrinogen are more strongly associated with colon (HR: 2.12; 95% CI: 1.21–3.73; Figure 2) than with rectum cancer (HR: 1.44; 95% CI: 0.59-3.53; Figure 2). Main results remained consistent after excluding *N* = 35 CRC cases that occurred during the first 12 months of follow-up (HR: 1.77; 95% CI: 0.99–3.17) (Supplementary Table S6) or in subgroup analyses stratified by age at CRC diagnosis (<60 years and ≥60 years) (Supplementary Table S7).

In addition, we examined the association between fibrinogen levels and CRC stratified by stage (I-II, III and IV) and grade (1, 2, and 3). In particular, elevated plasma levels of fibrinogen (≥ 400 mg/dL) were found associated mainly with stages III (HR: 4.16; 95% CI: 1.86–9.32) and IV (HR: 3.19; 95% CI: 1.25–8.11) and grade 3 (HR: 5.23; 95% CI: 1.98–13.85) (Supplementary Table S8). We repeated the analysis also excluding the first 12 months of follow-up, but data still remained consistent (Supplementary Table S8).

The investigation of the potential confounding effect of inflammatory and hemostasis biomarkers on the association between fibrinogen and CRC showed that (a) high sensitive-C reactive protein (hs-CRP) reduced the magnitude of the association and (b) the inclusion of hemostasis biomarkers (D-dimer or tissue plasminogen activator (tPA)) moderately increased the previous results (Table 3). However, when we fitted a model including all these factors, the findings remained consistent (HR: 1.83; 95% CI: 1.04–3.23).

**Table 3:** HRs (95% CI) for developing colorectal cancer according to levels of fibrinogen < or ≥ 400 mg/dL adding inflammatory and hemostasis biomarkers in the model.

	**Fibrinogen**		
	**<400 mg/dL**	**≥400 mg/dL**	***p*-value**
**Events/Sub-cohort**	100/1,166	26/124	
**HR** ^a^	Reference	1.91 (1.15–3.17)	0.012
**HR**^a^ **+** **hs-CRP**	Reference	1.74 (1.04–3.09)	0.03
**HR**^a^ **+** **D-dimer**	Reference	1.98 (1.17–3.35)	0.011
**HR**^a^ **+** **tPA**	Reference	2.04 (1.23–3.40)	0.0059
**HR** ^b^	Reference	1.83 (1.04–3.23)	0.036

hs-CRP, high sensitive C-reactive protein; tPA, tissue plasminogen activator.

a Model 2: adjusted for age, sex, CRC family history, income, physical activity, diabetes medication and hypercholesterolemia.

b Model 3: model 2 plus hs-CRP, D-dimer and tPA.

Further investigation on the influence of other biomarkers reported that a) glucose, HDL, aspartate aminotransferase (AST) and alanine transferase (ALT) did not affect the association between fibrinogen and CRC and b) the magnitude of the association was slightly reduced when uric acid was included in the final model (Supplementary Table S9). The association of fibrinogen with CRC, still remained consistent (HR:1.72; 95% CI: 1.04–2.86) (Supplementary Table S9), when we fitted a further model including the most relevant biomarkers (glucose, HDL, uric acid, and ALT).

## Discussion

Our results, based on a case-cohort study nested in the Moli-sani cohort apparently cancer free at the recruitment, showed that fibrinogen levels ≥400 mg/dL were independently and positively associated with CRC, suggesting that this glycoprotein could be a potential biomarker for this type of cancer.

To reduce the likelihood of CRC status already in progress before the fibrinogen measurement, we repeated the main analysis excluding *N* = 35 CRC cases occurred during the first 12 months of follow-up and the findings remained consistent.

Stratification analyses for age classes and sex showed a most evident association in elderly and in women, but without a statistically significant interaction for both.

Main findings of the present work are in line with a recent study performed in a Danish general population, showing that high levels of fibrinogen (>11.9 μmol/L≈ 404 mg/dL) were associated with an increased risk of CRC (31). In their conclusions, Authors speculated that the potential role of fibrinogen in cancer_risk could be explained by its involvement in inflammatory responses, supporting the role of chronic low-grade inflammation as an underlying mechanism for cancer progression (31, 44).

We found that the association between fibrinogen levels and CRC, was independent from the potential confounding effect of other inflammatory (hs-CRP) and hemostasis (D-dimer or tPA) factors. Therefore, although chronic low-grade inflammation is thought to be the underlying mechanism explaining the association of fibrinogen with the higher hazard to develop CRC, our study emphasizes the notion that inflammation and hemostasis should be considered together. Consequently, due to its dual role in inflammation and hemostasis, fibrinogen, could be considered as an ideal candidate biomarker for the prediction to develop CRC.

Besides these hemostatic and inflammatory factors, we also explored the potential confounding effect of a large panel of biomarkers (uric acid, AST, ALT, glucose and HDL), reportedly involved in colorectal cancer risk (39–42). In our study, these markers only slightly modified the association between fibrinogen and CRC.

Much is known about the mutual connection among cancer progression and hemostatic system and, several enzymes of the latter have been found to predict poor outcomes in cancer patients (15, 18, 45). Although cancer (24–30) and CVD (21, 22) were traditionally considered as distinct disorders, today there is an increasing evidence reporting the shared pathophysiology and an overlapping risk factors profile between these two conditions, supporting the so-called “common soil hypothesis” (10–15, 34). Two previous studies from our group reported the predictive role of other two hemostatic biomarkers in a general population. The first one showed the predictive role of plasminogen activator inhibitor-1 for both CVD and the occurrence of breast and colorectal cancer in a European cohort (46); the second one observed that elevated levels of tPA, a fibrinolytic protein with a well-known role in CVD risk, is a potential predictor for breast cancer in Moli-sani women (47).

Colon cancer (CC) and rectal cancer (RC) are commonly regarded as a single tumor entity termed colorectal cancer (CRC). However, evidence has been accumulated in the last decades that these are different disorders as their topography, surgical challenge, complications, metastatic pattern and therapeutic approach are concerned (48, 49). Also in terms of epidemiology, CC and RC have varying incidence and a different sex distribution (48). Whether these two subtypes of cancer have also different predictive and prognostic biomarkers, to date, has been not yet investigated (48). In this context, we tried to investigate a different relationship among fibrinogen and the two distinct cancer subtypes. The positive association between high levels of fibrinogen and CRC was stronger, if not restricted to, in CC subtype, rather than in RC. Additionally, we also stratified the analyses by CRC stage and grade, confirming the main result for CRC stages III and IV, and with grade 3.

However, due to the paucity of CRC cases by subtype/stage/grade categorizations, further studies are required to confirm these observations.

### Strengths and limitations

The prospective design represents, above all, the main strength of the present work. In addition, the availability of a wide and heterogeneous panel of information allowed us the control for their possible confounding effect.

However, our findings should be viewed in light of some limitations linked to the fibrinogen measurements:

(i) for each participant, plasma sample were acquired only during the baseline visit, therefore, indications on possible variations on the long term are lacking; (ii) the measurements were performed on frozen samples thawed many years after their storage in a dedicated biobank. The latter limitation could theoretically influence the laboratory data but, freezing and long-term storing seems to have minimal effect on fibrinogen levels and it is irrelevant for clinical interpretation (37).

## Conclusion

Elevated plasma levels of fibrinogen (≥400 mg/dL) are related to an increased hazard of colorectal cancer in a general adult population apparently free from any cancer during the recruitment. This association, in particular was independent from the potential confounding effect of other inflammatory (hs-CRP) and hemostasis (D-dimer or tPA) factors. Our results add further support to the “common soil” hypothesis in the pathophysiology of cancer and CVD (10, 34).

## Data availability statement

The data underlying this article will be shared on reasonable request to the corresponding author. The data are stored in an institutional repository (https://repository.neuromed.it) and access is restricted by the ethical approvals and the legislation of the European Union.

## Ethics statement

The studies involving human participants were reviewed and approved by Catholic University Ethical Committee, Rome, Italy. The patients/participants provided their written informed consent to participate in this study.

## Author contributions

AF, LI, MBD, and MM contributed to the concept and design of the work and interpretation of data. SG and LR performed the fibrinogen measurements. SC, ADiC, ADeC, SG, LR, MM, and TP managed data collection. SC and RP wrote the paper and performed the statistical analyses. CC, MBD, AF, GdG, and LI originally inspired the research and critically reviewed the manuscript. All authors contributed to the article and approved the submitted version.

## Funding

The analyses conducted in this study were partially supported by the HYPERCAN Study (AIRC 5xMILLE no. 12237) and by the Italian Ministry of Health (Ricerca Corrente 2022–2024).

The enrolment phase of the Moli-sani Study was supported by unrestricted research grants from the Pfizer Foundation (Rome, Italy), the Italian Ministry of University and Research (MIUR, Rome, Italy)—Programma Triennale di Ricerca, Decreto no. 1588, and Instrumentation Laboratory, Milan, Italy. The follow-up phase of the Moli-sani Study (assessment of incident cases) was partially supported by AIRC 5xMILLE (HYPERCAN Study, no. 12237) and the Italian Ministry of Health (PI GdG, CoPI SC; grant no. RF-2018-12367074). Funders had no role in study design, collection, analysis, interpretation of data, writing of the manuscript, and decision to submit this article for publication.

## Conflict of interest

The authors declare that the research was conducted in the absence of any commercial or financial relationships that could be construed as a potential conflict of interest.

## Publisher's note

All claims expressed in this article are solely those of the authors and do not necessarily represent those of their affiliated organizations, or those of the publisher, the editors and the reviewers. Any product that may be evaluated in this article, or claim that may be made by its manufacturer, is not guaranteed or endorsed by the publisher.
