# Supplementary material for: Fibrinogen levels in relation to colorectal cancer onset: A nested case-cohort study from the Moli-sani cohort
Source: Front Cardiovasc Med. 2022 Oct 13;9:1009926. doi: 10.3389/fcvm.2022.1009926 (PMC9606318; doi:10.3389/fcvm.2022.1009926)
Supplement: Supplementary file 1 [file Data_Sheet_1.docx]

**Supplementary file**

*Definition of baseline characteristics*

CRC family history, lifestyle and dietary habits and medical history and drug therapy were collected during the baseline visit. Blood pressure was measured by an automatic device (OMRON-HEM-705CP) three times on the non-dominant arm and the average of the last two values was taken as the BP. Measurements were made in a quiet room with comfortable temperature with the participants lying down for at least 5 minutes. Hypertension was described as systolic blood pressure ≥ 140 mm Hg or diastolic blood pressure ≥ 90mm Hg, or pharmacological treatment.

Diabetes diagnosis were ascertained considering blood glucose level ≥ 126 mg/dL or considering individual using of pharmacological treatment.

Hypercholesterolemia was identified considering total cholesterol values higher than 240 mg/dL or pharmacological treatment.

Subjects were classified as “non-smokers” if they had smoked less than 100 cigarettes in their lifetime, or they had never smoked cigarettes, or if they had smoked cigarettes in the past and had stopped smoking for at least 1 year, and “current smokers” if reported having smoked at least 100 cigarettes in their lifetime and still smoked or had quit smoking within the preceding year.

Educational attainment was based on the highest qualification attained and categorized as low (up to lower secondary school; approximately ≤ 8 years of study) or high (upper secondary education or higher; approximately ≥ 9 years of study). Household income was a three-level variable (≤ 40,000; >40,000 Euros/year), with missing values collapsed into a non respondent category. Physical activity was assessed by a structured questionnaire (24 questions on working time, leisure time, weekly walking, and sports participation) and expressed as daily energy expenditure in metabolic equivalent task-hours [1].

Body weight and height were measured on a standard beam balance scale with an attached ruler with subjects wearing no shoes and only light indoor clothing, BMI was calculated as kg/m^2^.

Obesity was defined by a BMI of equal to or greater than 30 kg/m^2^.

Waist circumference was measured according to the NIH, Heart, Lung, and Blood guidelines [2]. Abdominal obesity was defined by a waist circumference of greater than 102 cm for men and 88 cm for women. Adherence to the Mediterranean diet was evaluated by the Mediterranean Diet Score (MDS; range 0 to 9) developed by Trichopoulou et al. [3]. The average volume of alcohol (g/day) consumed during the year before enrolment was assessed by the validated Italian EPIC food frequency questionnaire, complemented by specific supplementary questions [4].

*Laboratory Procedures*

Serum lipids (HDL-cholesterol, triglycerides) and blood glucose were assayed by enzymatic reaction methods using an automatic analyzer (ILab 350, Instrumentation Laboratory [IL], Milan, Italy). Quality control for lipids and glucose was performed using a commercial standard (Ser 1 and Ser 2) provided by the IL and an in-house serum standard pool. The coefficients of variability (CVs) were respectively 3.2, 3, and 4.5% for HDL-cholesterol; 5.2, 5.3, and 5% for triglycerides; and 4.7, 4.1, and 3.9% for blood glucose.

High-sensitivity C-reactive protein (hs-CRP) was measured in fresh serum samples by a particle-enhanced immunoturbidimetric assay (ILab 350; IL). Quality control for hs-CRP was performed using an in-house serum pool and internal laboratory standard; interday CVs for hs-CRP were 5.5 and 4.2%, respectively.

D-dimer levels were measured on fresh citrated plasma by an automated latex-enhanced immunoassay (HemosIL-IL, Milan, Italy). Quality control was maintained using an internal laboratory standard in-house plasma pool. Inter and intra-day variability coefficients were 5.4% and 7.6%, respectively [6].

Uric Acid, Aspartate aminotransferase (ALT) and Alanine transferase (AST) were measured by colorimetric enzyme kit and albumin by colorimetric assay. All tests were performed by an automatic analyzer (ILab ARIES; Instrumentation Laboratory, Milan, Italy). Quality control was assured using commercial (high and low) laboratory standards. Coefficients of variation (CV) for high- and low-level external standards were 5.3% and 5.0% for Uric Acid; were 3.9% and 3.6% for ALT; 6.0% and 4.5% for AST; and 3.4% and 3.6% for albumin.

Additionally, in the framework of the European BiomarCaRE project, insulin and markers of renal function (cystatin C, creatinine), were measured on frozen serum samples in 2014 [7].

Tissue plasminogen activator (tPA) was measured by enzyme linked immunosorbent assay (ELISA) with a commercial kit (Zymutest tPA antigen, HYPHEN BioMed, France). Quality controls included high and low plasma samples, provided by the kit, and a home-made normal pool plasma. The intra- and interassay CVs were 6 and 7%, respectively [8].

**References**

1. Ainsworth BE, Haskell WL, Whitt MC, et al. Compendium of physical activities: an update of activity codes and MET intensities. Med Sci Sports Exerc. 2000;32(9 Suppl):S498-S504.
2. Janssen I, Katzmarzyk PT, Ross R. Body mass index, waist circumference, and health risk: evidence in support of current national institutes of health guidelines. Arch Intern Med 2002; 162:2074e9
3. Trichopoulou A, Costacou T, Bamia C, Trichopoulos D. Adherence to a Mediterranean diet and survival in a Greek population. N Engl J Med 2003;348:2599e608
4. Costanzo S, Mukamal KJ, Di Castelnuovo A, et al; Moli-sani Study Investigators. Alcohol consumption and hospitalization burden in an adult Italian population: prospective results from the Moli-sani study. Addiction 2019;114(04):636–650
5. Santimone I, Di Castelnuovo A, De Curtis A, et al; MOLI-SANI Project Investigators. White blood cell count, sex and age are major determinants of heterogeneity of platelet indices in an adult general population: results from the MOLI-SANI project. Haematologica 2011;96(08):1180–1188
6. Di Castelnuovo A, de Curtis A, Costanzo S, et al. Association of D-dimer levels with all-cause mortality in a healthy adult population: findings from the MOLI-SANI study. Haematologica. 2013;98(9):1476-1480.
7. Zeller T, Hughes M, Tuovinen T, et al. BiomarCaRE: rationale and design of the European BiomarCaRE project including 300,000 participants from 13 European countries. Eur J Epidemiol 2014;29 (10):777–790
8. Costanzo S, Parisi R, De Curtis A, et al. Tissue Plasminogen Activator Levels and Risk of Breast Cancer in a Case-Cohort Study on Italian Women: Results from the Moli-sani Study. Thromb Haemost. 2021;121(4):449-456.

**Table S1.** Baseline characteristic in the subcohort (N=1,290) and cases (N= 126), according to levels of plasma fibrinogen < or ≥ 400 mg/dL

|  | **Subcohort**  **N 1,290** | | | **Cases**  **N 126** | | |
| --- | --- | --- | --- | --- | --- | --- |
|  | **Fibrinogen**  **< 400 mg/dL** | **Fibrinogen**  **≥ 400 mg/dL** | **P value** | **Fibrinogen**  **<400 mg/dL** | **Fibrinogen**  **≥400 mg/dL** | **P value** |
| **SBP,** ***mm Hg*** | 140.1 ± 21.4 | 145.9 ± 20.6 | 0.50 | 149.1 ± 21.0 | 148.5 ±16.1 | 0.60 |
| **DBP, *mm Hg*** | 82.1 ± 9.8 | 83.5 ± 9.6 | 0.32 | 83.4 ± 9.4 | 81.8 ± 8.7 | 0.70 |
| **Glucose*, mg/dL*** | 96.0 (88.0-106.0) | 101.0 (89.0-112.0) | 0.15 | 100.0 (90.0-116.5) | 104.5 (94.0-120.0) | 0.47 |
| **Insulin, *pmol/L*** | 50.3 (35.9-66.5) | 59.5 (46.4-86.2) | <.0001 | 47.1 (33.9-62.7) | 60.7 (46.0-74.5) | 0.060 |
| **Triglycerides, *mg/dL*** | 105.0 (78.0-146.0) | 113.0 (80.5-153.5) | 0.20 | 113.5 (88.0-158.0) | 152.5(90.0-191.0) | 0.063 |
| **HDL-cholesterol *mg/dL*** | 58.6 ±15.0 | 53.6 ±14.8 | 0.0002 | 54.6 ± 12.4 | 49.4 ± 13.9 | 0.031 |
| **hs-CRP, *mg/L*** | 1.38 (0.71-2.57) | 3.49 (1.81-7.44) | <.0001 | 1.6 (0.74-3.43) | 5.4 (1.7-13.7) | <.0001 |
| **D-dimer, *ng/L*** | 188.0 (144.0-222.0) | 203.0 (163.0-365.0) | <.0001 | 172.0 (101.5-250.0) | 198.0 (136.0-352.0) | 0.54 |
| **tPA, *ng/mL*** | 8.8 (5.6-12.2) | 10.3 (6.9-14.9) | 0.0048 | 8.6 (6.03-11.1) | 11.5 (8.3-14.0) | 0.047 |
| **Creatinine, *mg/dL*** | 0.77 (0.70-0.87) | 0.79 (0.69-0.91) | 0.30 | 0.84 (0.76-0.97) | 0.87 (0.77-1.01) | 0.90 |
| **Cystatin C, *mg/dL*** | 0.94 (0.83-1.05) | 1.02 (0.91-1.15) | 0.0005 | 1.03 (0.91-1.18) | 1.14 (0.94-1.34) | 0.14 |
| **Albumin, *g/dL*** | 4.2 ± 0.3 | 4.1 ± 0.3 | 0.45 | 4.1 ± 0.3 | 4.0 ± 0.4 | 0.37 |
| **AST, *U/L*** | 23.0 (20.0-27.0) | 23.5 (20.0-27.0) | 0.13 | 23.5 (21.0-26.5) | 21.5 ( 19.0-24.0) | 0.0083 |
| **ALT, *U/L*** | 19.00 (15.0-27.0) | 21.0 (16.0-27.0) | 0.19 | 19.5 (15.0-24.5) | 17.0 ( 14.0- 19.0) | 0.044 |
| **Uric Acid, *mg/dL*** | 5.2 ± 1.4 | 5.6 ± 1.6 | 0.016 | 5.7 ± 1.6 | 6.5 ± 1.9 | 0.032 |
| **Caloric Intake *kcal/day*** | 2138.3 ± 670.2 | 1992.3 ± 5178.7 | 0.10 | 2048.3 ± 658.6 | 2000.7 ± 692.4 | 0.88 |
| **Med Diet Score** | 4.3 ± 1.6 | 4.6 ± 1.4 | 0.21 | 4.9 ±1.7 | 4.5 ± 1.5 | 0.39 |
| **Alcohol intake *g/day*** | 16.2 ± 23.5 | 13.1 ± 19.9 | 0.018 | 22.2 ± 27.2 | 16.1 ± 19.1 | 0.33 |
| ***Food group consumption*** |  |  |  |  |  |  |
| **Lipids *g/day*** | 77.6 ± 26.2 | 73.1 ± 23.3 | 0.41 | 71.1 ± 23.8 | 67.3 ± 23.9 | 0.82 |
| **Red meat *g/day*** | 45.7 ± 24.4 | 44.6 ± 23.1 | 0.99 | 43.3 ± 28.8 | 38.6 ± 17.6 | 0.69 |
| **Processed meats *g/day*** | 29.1 ± 20.5 | 25.0 ± 18.7 | 0.22 | 24.2 ± 17.2 | 20.5 ± 14.9 | 0.63 |
| **All red meats *g/day*** | 74.8 ± 37.3 | 69.6 ± 34.9 | 0.49 | 67.5 ± 35.8 | 59.0 ± 25.7 | 0.57 |

**Abbreviations:** SBP, systolic blood pressure; DBP, diastolic blood pressure; HDL, high density lipoprotein;

hs-CRP high sensitive C-reactive protein; tPA, tissue plasminogen activator; AST, aspartate aminotransferase; ALT, alanine transaminase.

**Note**: Values are reported as means with standard deviation. Median values and IQR are reported for glucose, insulin, triglycerides, hs-CRP, D-dimer, tPA, creatinine, cystatin C, AST and ALT. P values are adjusted for age and sex.

**Table S2.** HRs (95% CI) for developing colorectal cancer in relation to fibrinogen levels.

|  | **Quartiles of Fibrinogen *(mg/dL)*** | | | | **P trend** | **Continuous**  **(*for every SD increase of log Fibrinogen*)** |
| --- | --- | --- | --- | --- | --- | --- |
| **Sex specific range:**  **Women**  **Men** | **I**  **59-252**  **55-238** | **II**  **253-301**  **239-280** | **III**  **302-350**  **281-336** | **IV**  **>350**  **>336** |  |  |
| **Events/Subcohort** | 27/323 | 20/323 | 37/326 | 42/318 |  |  |
| **HR crude** | Reference | 0.62 (0.34-1.15) | 1.00 (0.57-1.73) | 1.02 (0.59-1.74) | 0.52 | 1.12 (0.90-1.40) |
| **HR^a^** | Reference | 0.59 (0.32-1.09) | 0.98 (0.56-1.72) | 1.03 (0.60-1.77) | 0.45 | 1.16 (0.93-1.43) |
| **HR^b^** | Reference | 0.65 (0.34-1.24) | 1.14 (0.63-2.06) | 1.14 (0.65-2.00) | 0.29 | 1.19 (0.96-1.49) |

**^a^Model 1**: adjusted for age and sex;

**^b^Model 2**: adjusted for age, sex, CRC family history, income, physical activity, diabetes medication and hypercholesterolemia

**Table S3.** HRs (95% CI) for developing colorectal cancer according to levels of fibrinogen < or ≥ 400 mg/dL, including one by one demographic, lifestyle and clinical variables in the model adjusted for age and sex.

|  | **Fibrinogen** | |  |
| --- | --- | --- | --- |
|  | **<400 mg/dL** | **≥400 mg/dL** | **P value** |
| **Events/Subcohort** | 100/1,166 | 26/124 |  |
| **HR crude** | Reference | 1.77 (1.09-2.85) | 0.020 |
| **HR^a^** | Reference | 1.81 (1.12-2.92) | 0.015 |
| **HR^a^** + family history | Reference | 1.85 (1.15-2.99) | 0.012 |
| **HR^a^** + residence | Reference | 1.80 (1.11-2.89) | 0.017 |
| **HR^a^** + education | Reference | 1.83 (1.14-2.95) | 0.013 |
| **HR^a^** + income | Reference | 1.87 (1.15-3.03) | 0.011 |
| **HR^a^** + BMI | Reference | 1.87 (1.15-3.05) | 0.012 |
| **HR^a^** + obesity | Reference | 1.83 (1.12-2.99) | 0.015 |
| **HR^a^** + current smoking | Reference | 1.81 (1.12-2.92) | 0.016 |
| **HR^a^** + physical activity | Reference | 1.88 (1.16-3.07) | 0.011 |
| **HR^a^** + history of CVD | Reference | 1.86 (1.20-2.88) | 0.0055 |
| **HR^a^** + diabetes medication | Reference | 1.68 (1.02-2.77) | 0.043 |
| **HR^a^** + hypercholesterolemia | Reference | 1.85 (1.15-3.00) | 0.012 |
| **HR^a^** + hypertension | Reference | 1.83 (1.13-2.95) | 0.014 |

^a^Model adjusted for age and sex.

**Table S4.**  ORs (95% CI) for colorectal cancer according to levels of fibrinogen < or ≥ 400 mg/dL Case-control approach.

|  | **Fibrinogen** | |  | |  |
| --- | --- | --- | --- | --- | --- |
|  | **<400 mg/dL** | **≥400 mg/dL** | | **P value** | |
| **Case/Control** | 100/1,160 | 26/122 | |  | |
| **OR crude** | Reference | 2.47 (1.55-3.96) | | 0.0002 | |
| **OR^a^** | Reference | 1.90 (1.16-3.10) | | 0.011 | |
| **OR^a^** + family history | Reference | 1.92 (1.17-3.14) | | 0.0096 | |
| **OR^a^** + residence | Reference | 1.85 (1.13-3.03) | | 0.015 | |
| **OR^a^** + education | Reference | 1.94 (1.18-3.17) | | 0.0086 | |
| **OR^a^** + income | Reference | 1.92 (1.17-3.15) | | 0.0099 | |
| **OR^a^** +BMI | Reference | 1.95 (1.19-3.22) | | 0.0087 | |
| **OR^a^** + obesity | Reference | 1.93 (1.18-3.19) | | 0.0095 | |
| **OR^a^** + current smoking | Reference | 1.89 (1.16-3.10) | | 0.011 | |
| **OR^a^** + physical activity | Reference | 1.98 (1.21-3.24) | | 0.0070 | |
| **OR^a^** + history of CVD | Reference | 1.92 (1.17-3.15) | | 0.010 | |
| **OR^a^** + diabetes medication | Reference | 1.79 (1.08-2.96) | | 0.0228 | |
| **OR^a^** + hypercholesterolemia | Reference | 1.92 (1.17-3.14) | | 0.0096 | |
| **OR^a^** + hypertension | Reference | 1.93 (1.18-3.15) | | 0.0093 | |
| **OR^b^** | Reference | 1.93 (1.16-3.21) | | 0.011 | |

**^a^Model 1**: adjusted for age and sex.

**^b^Model 2**: adjusted for age, sex, CRC family history, income, physical activity, diabetes medication and hypercholesterolemia.

**Table S5.** Sensitivity analyses stratified by age and sex according to levels of fibrinogen < or ≥ 400 mg/dL

|  | **Fibrinogen** | |  | **P for**  **interaction** |
| --- | --- | --- | --- | --- |
|  | **< 400 mg/dL** | **≥ 400 mg/dL** | **P value** |  |
|  | **Age 35-65 years** | | | 0.26 |
| **Events/Subcohort** | 51/922 | 10/83 |  |  |
| **HR^a^** | Reference | 1.43 (0.65-3.17) | 0.38 |  |
|  | **Age ≥ 65 years** | | |  |
| **Events/Subcohort** | 49/244 | 16/41 |  |  |
| **HR^a^** | Reference | 2.30 (1.10-4.81) | 0.027 |  |
|  | **Women** | | | 0.72 |
| **Events/Subcohort** | 36/642 | 11/63 |  |  |
| **HR^b^** | Reference | 2.28 (1.08-4.81) | 0.031 |  |
| **HR^c^** | Reference | 2.24 (1.06-4.70) | 0.034 |  |
|  | **Men** | | |  |
| **Events/Subcohort** | 64/524 | 15/61 |  |  |
| **HR^b^** | Reference | 1.84 (0.90-3.78) | 0.096 |  |

**^a^Model** adjusted for age, sex, CRC family history, income, physical activity diabetes medication and hypercholesterolemia.

**^b^Model** adjusted for age, CRC family history, income, physical activity diabetes medication and hypercholesterolemia.

**^c^Model** adjusted for age, menopausal status, CRC family history, income, physical activity, diabetes medication and hypercholesterolemia.

**Table S6.** Sensitivity analyses for developing colorectal cancer according to levels of fibrinogen < or ≥ 400 mg/dL, excluding cases occurred in the first 12 months of follow-up.

|  | **Fibrinogen** | |  |
| --- | --- | --- | --- |
|  | **< 400 mg/dL** | **≥ 400 mg/dL** | **P value** |
| **Events/Subcohort** | 73/1,163 | 18/123 |  |
| **HR crude** | Reference | 1.68 (0.97-2.91) | 0.067 |
| **HR^a^** | Reference | 1.79 (1.02-3.12) | 0.041 |
| **HR^b^** | Reference | 1.77 (0.99-3.17) | 0.056 |

**^a^Model 1**: adjusted for age and sex;

**^b^Model 2**: adjusted for age, sex, CRC family history, income, physical activity, diabetes medication and hypercholesterolemia.

**Table S7.** Sensitivity analyses stratified by age at colorectal cancer (CRC) diagnosis according to levels of fibrinogen < or ≥ 400 mg/dL

|  | **Fibrinogen** | |  |
| --- | --- | --- | --- |
|  | **< 400 mg/dL** | **≥ 400 mg/dL** | **P value** |
|  | **Age at CRC Diagnosis < 60 years** | |  |
| **Events/Subcohort** | **29/1,166** | **5/124** |  |
| **HR^a^** | Reference | 1.87 (0.69-5.08) | 0.22 |
| **HR^b^** | Reference | 2.00 (0.70-5.72) | 0.19 |
|  | **Age at CRC Diagnosis ≥ 60 years** | |  |
| **Events/Subcohort** | **71/1,166** | **21/124** |  |
| **HR^a^** | Reference | 1.80 (1.05-3.10) | 0.033 |
| **HR^b^** | Reference | 1.94 (1.06-3.57) | 0.033 |

**^a^Model 1**: adjusted for age and sex;

**^b^Model 2**: adjusted for age, sex, CRC family history, income, physical activity, diabetes medication and hypercholesterolemia.

**Table S8.** Sensitivity analyses stratified by CRC stage and grade according to levels of fibrinogen < or ≥ 400 mg/dL.

|  | **Whole Sample** | | | **After excluding the first 12 months of follow-up** | | | |
| --- | --- | --- | --- | --- | --- | --- | --- |
|  | **Fibrinogen** | | |  | **Fibrinogen** | | |
|  | **< 400 mg/dL** | **≥ 400 mg/dL** | **P value** |  | **< 400 mg/dL** | **≥ 400 mg/dL** | **P value** |
| **CRC STAGE** | **Cancer Stage I-II** | | |  | **Cancer Stage I-II** | | |
| **Events/Subcohort** | 47/1,166 | 7/124 |  | **Events/Subcohort** | 33/1,163 | 4/123 |  |
| **HR^a^** | Reference | 1.06 (0.47-2.40) | 0.88 | **HR^a^** | Reference | 0.91 (0.31-2.64) | 0.86 |
| **HR^b^** | Reference | 1.07 (0.45-2.54) | 0.88 | **HR^b^** | Reference | 0.78 (0.25-2.48) | 0.67 |
|  | **Cancer Stage III** | | |  | **Cancer Stage III** | | |
| **Events/Subcohort** | 19/1,166 | 9/124 |  | **Events/Subcohort** | 16/1,163 | 7/123 |  |
| **HR^a^** | Reference | 3.32 (1.51-7.30) | 0.0028 | **HR^a^** | Reference | 3.08 (1.26-7.52) | 0.013 |
| **HR^b^** | Reference | 4.16 (1.86-9.32) | 0.0005 | **HR^b^** | Reference | 3.98 (1.75-9.08) | 0.0010 |
|  | **Cancer Stage IV** | | |  | **Cancer Stage IV** | | |
| **Events/Subcohort** | 17/1,166 | 7/124 |  | **Events/Subcohort** | 13/1,163 | 5/123 |  |
| **HR^a^** | Reference | 2.89 (1.19-7.00) | 0.019 | **HR^a^** | Reference | 2.85 (1.01-8.03) | 0.047 |
| **HR^b^** | Reference | 3.19 (1.25-8.11) | 0.015 | **HR^b^** | Reference | 3.14 (1.11-8.87) | 0.031 |
| **CRC GRADE** | **Cancer Grade 1** | | |  | **Cancer Grade 1** | | |
| **Events/Subcohort** | 10/1,166 | 3/124 |  | **Events/Subcohort** | 10/1,163 | 2/123 |  |
| **HR^a^** | Reference | 2.15 (0.61-7.63) | 0.24 | **HR^a^** | Reference | 1.42 (0.32-6.26) | 0.64 |
| **HR^b^** | Reference | 1.93 (0.50-7.42) | 0.34 | **HR^b^** | Reference | 1.25 (0.25-6.24) | 0.79 |
|  | **Cancer Grade 2** | | |  | **Cancer Grade 2** | | |
| **Events/Subcohort** | 55/1,166 | 13/124 |  | **Events/Subcohort** | 39/1,163 | 9/123 |  |
| **HR^a^** | Reference | 1.68 (0.88-3.20) | 0.11 | **HR^a^** | Reference | 1.72 (0.79-3.73) | 0.17 |
| **HR^b^** | Reference | 1.74 (0.85-3.55) | 0.13 | **HR^b^** | Reference | 1.57 (0.68-3.64) | 0.29 |
|  | **Cancer Grade 3** | | |  | **Cancer Grade 3** | | |
| **Events/Subcohort** | 8/1,166 | 7/124 |  | **Events/Subcohort** | 7/1,163 | 6/123 |  |
| **HR^a^** | Reference | 5.47 (2.13-14.07) | 0.0004 | **HR^a^** | Reference | 5.56 (2.01-15.39) | 0.0009 |
| **HR^b^** | Reference | 5.23 (1.98-13.85) | 0.0009 | **HR^b^** | Reference | 5.23 (1.88-14.52) | 0.0015 |

**^a^Model 1**: adjusted for age and sex;

**^b^Model 2**: adjusted for age, sex, CRC family history, income, physical activity, diabetes medication and hypercholesterolemia.

**Table S9.** HRs (95% CI) for developing colorectal cancer according to levels of fibrinogen < or ≥ 400 mg/dL including biomarkers in the model.

|  | **Fibrinogen** | |  |
| --- | --- | --- | --- |
|  | **< 400 mg/dL** | **≥ 400 mg/dL** | **P value** |
| **Events/Subcohort** | 100/1,166 | 26/124 |  |
| **HR^a^** | Reference | 1.91 (1.15-3.17) | 0.012 |
| **HR_2_ + Glucose** | Reference | 1.89 (1.14-3.13) | 0.014 |
| **HR_2_ + HDL** | Reference | 1.84 (1.12-3.04) | 0.017 |
| **HR_2_ + Uric acid** | Reference | 1.80 (1.08-3.01) | 0.024 |
| **HR_2_ + AST** | Reference | 1.93 (1.16-3.20) | 0.011 |
| **HR_2_ + ALT** | Reference | 1.93 (1.17-3.20) | 0.011 |
| **HR^b^** | Reference | 1.72 (1.04-2.86) | 0.035 |

**Abbreviation:** HDL, High-Density Lipoprotein; AST, Aspartate aminotransferase; ALT, Alanine aminotransferase.

**^a^ Model 2**: adjusted for age, sex, CRC family history, income, physical activity, diabetes medication and hypercholesterolemia.

**^b^Model 3:** model 2 plus glucose, HDL, Uric acid and ALT.

**Appendix S1: Moli-sani Study Investigators**

The enrolment phase of the Moli-sani Study was conducted at the Research Laboratories of the Catholic University in Campobasso (Italy), the follow up of the Moli-sani cohort is being conducted at the Department of Epidemiology and Prevention of the IRCCS Neuromed, Pozzilli, Italy.

**Steering Committee**: Licia Iacoviello*°(Chairperson), Giovanni de Gaetano* and Maria Benedetta Donati*.

**Scientific Secretariat**: Marialaura Bonaccio*, Americo Bonanni*, Chiara Cerletti*, Simona Costanzo*, Amalia De Curtis*, Augusto Di Castelnuovo§, Alessandro Gialluisi*°, Francesco Gianfagna°§, Mariarosaria Persichillo*, Teresa Di Prospero* (Secretary).

**Safety and Ethical Committee**: Jos Vermylen (Catholic University, Leuven, Belgio) (Chairperson), Renzo Pegoraro (Pontificia Accademia per la Vita, Roma, Italy), Antonio Spagnolo (Catholic University, Roma, Italy).

**External Event Adjudicating Committee**: Deodato Assanelli (Brescia, Italy), Livia Rago (Campobasso, Italy).

**Baseline and Follow-up Data Management**: Simona Costanzo* (Coordinator), Marco Olivieri (Campobasso, Italy), Teresa Panzera*.

**Data Analysis**: Augusto Di Castelnuovo§ (Coordinator), Marialaura Bonaccio*, Simona Costanzo*, Simona Esposito*, Alessandro Gialluisi*°, Francesco Gianfagna°§, Sabatino Orlandi*, Emilia Ruggiero*, Alfonsina Tirozzi*.

**Biobank, Molecular and Genetic Laboratory**: Amalia De Curtis* (Coordinator), Sara Magnacca§, Fabrizia Noro*, Alfonsina Tirozzi*.

**Recruitment Staff**: Mariarosaria Persichillo* (Coordinator), Francesca Bracone*, Teresa Panzera*.

**Communication and Press Office**: Americo Bonanni*.

**Regional Institutions**: Direzione Generale per la Salute - Regione Molise; Azienda Sanitaria Regionale del Molise (ASReM, Italy); Agenzia Regionale per la Protezione Ambientale del Molise (ARPA Molise, Italy); Molise Dati Spa (Campobasso, Italy); Offices of vital statistics of the Molise region.

**Hospitals**: Presidi Ospedalieri ASReM: Ospedale A. Cardarelli – Campobasso, Ospedale F. Veneziale – Isernia, Ospedale San Timoteo - Termoli (CB), Ospedale Ss. Rosario - Venafro (IS), Ospedale Vietri – Larino (CB), Ospedale San Francesco Caracciolo - Agnone (IS); Casa di Cura Villa Maria - Campobasso; Ospedale Gemelli Molise - Campobasso; IRCCS Neuromed - Pozzilli (IS).

*Department of Epidemiology and Prevention, IRCCS Neuromed, Pozzilli, Italy

°Department of Medicine and Surgery, University of Insubria, Varese, Italy

§Mediterranea Cardiocentro, Napoli, Italy

*Moli-sani Study Past Investigators are available at https://www.moli-sani.org/?page_id=173*
